# CHIME: a novel HLA eplet model associates with non-relapse mortality in haploidentical hematopoietic cell transplant with post-transplant cyclophosphamide

**DOI:** 10.1038/s41409-026-02954-6

**Published:** 2026-07-04

**Authors:** Michael Carter, Sunmin Park, Michiko Taniguchi, Salman Otoukesh, Dongyun Yang, Ping Rao, Shukaib Arslan, Geoffrey Shouse, Haris Ali, Ketevan Gendzekhadze, Monzr M. Al Malki

**Affiliations:** 1 Histocompatibility (HLA) & Immunogenetics Laboratory, City of Hope, Duarte, CA USA; 2 Department of Hematology and Hematopoietic Cell Transplantation, City of Hope National Medical Center, Duarte, CA USA; 3 Department of Pathology and Immunology, Washington University, St. Louis, MO USA; 4 Department of Computational and Quantitative Medicine, City of Hope Beckman Research Institute, Duarte, CA USA; 5 Department of Internal Medicine, Harold C. Simmons Comprehensive Cancer Center, UT Southwestern Medical Center, Dallas, TX USA

**Keywords:** Bone marrow transplantation, Haematological cancer, Allotransplantation

## Abstract

The importance of HLA mismatch in haploidentical hematopoietic cell transplantation (HaploHCT) with post-transplant cyclophosphamide (PTCy) is debated. If HLA mismatch does impact outcomes, a molecular mismatch model could predict transplant outcomes more effectively than allele mismatch. In this retrospective study, we quantified molecular HLA disparity using eplet mismatch for 265 patient-donor pairs undergoing HaploHCT with PTCy for hematologic malignancy. We discovered an interaction among eplet mismatch (EpMM), HLA class, and mismatch vector that was associated with clinical outcomes. This interaction was termed Class-wise HLA Imbalance of Mismatched Eplets (CHIME), which comprised risk scores of 0, 1, and 2. The CHIME score retained association with non-relapse mortality (CHIME 1 vs. 0, HR 2.85 (1.10–7.37); CHIME 2 vs. 0, HR 3.63 (1.36–9.68), adjusted *p* = 0.046) and severe aGvHD grade III-IV (CHIME 1 HR 7.82 (1.03–59.22); CHIME 2 HR 8.08 (1.01–64.63), adjusted *p* = 0.046) after adjustment for transplant-related variables. The CHIME score, when combined with CD3 cell dose, correlated with higher rates of cytokine release syndrome (CRS). The CHIME model may improve clinical outcome prediction over HLA antigen or allele mismatch.

## Introduction

Haploidentical hematopoietic cell transplantation (HaploHCT) with post-transplant cyclophosphamide (PTCy) has improved access to HCT for patients who traditionally have a lower likelihood of a suitable matched donor or need urgent transplant due to high-risk disease. In the setting of unrelated donor transplantation, increased HLA mismatch has been associated with inferior survival and treatment-related mortality [1, 2]. In contrast, early studies of HaploHCT with PTCy found that HLA mismatch was not detrimental, and possibly beneficial [3]. Recent multicenter studies found no significant associations between antigen level mismatch models and patient outcomes [4, 5]. However, some authors have reported individual or cumulative HLA mismatches associated with various clinical endpoints [3, 6–10].

Molecular-level HLA mismatch models have also been reported in HaploHCT [11–15]. Eplets are one such model: distinct configurations of amino acid polymorphisms in close proximity on the mature antigen that could elicit an immune response [16]. Eplet mismatch (EpMM) calculators have been created [17], along with an online reference database [18]. Rimando et al. reported initially that higher EpMM in class II in the GvH vector in HaploHCT was associated with decreased relapse incidence and improved relapse-free survival without effects on overall survival [19]. In a separate, single institution-study of predominantly bone marrow graft haploHCT, Zou et al. reported significant associations of HLA-A, B and overall class I EpMM with survival and severe aGvHD [20]. However, a subsequent analysis of a CIBMTR cohort only found an association with HLA-DRB1 EpMM and delayed engraftment, similar to Rimando et al., but in the GvH vector instead of HvG [21]. While risk models have been developed for allele-level mismatches and transplant characteristics [9], a molecular mismatch risk model has not been validated.

In this study, we evaluated the impact of HLA mismatch on outcomes in 265 patients who underwent HaploHCT with PTCy for hematologic malignancy at our institution. We hypothesized that the effects of HLA mismatch on transplant outcomes would be better predicted by a more discretely quantifiable model than simple allele mismatch counts. The availability of full HLA high-resolution genotyping for this cohort allowed complete calculation of molecular mismatch profiles with minimal inferences. Although the initial EpMM calculation was not strongly associated with transplant outcomes, we identified a novel interaction model of EpMM between HLA Class I and Class II. Here, we present our algorithm (CHIME), which incorporates EpMM level, HLA class, and mismatch vector, and is associated with several transplant outcomes in our cohort.

## Methods

### Patients and eligibility

We retrospectively analyzed 265 patients. Inclusion criteria were first HaploHCT with PTCy-based GVHD prophylaxis for hematologic malignancies at City of Hope (2011-2020), with complete (11-loci) two-field genotyping. The study was IRB-approved with a waiver of consent. Diagnoses were classified per WHO criteria; HCT-CI [22] and disease risk index (DRI) were calculated as described [23]. CRS within 7 days post-transplant was graded using ASTCT criteria [24].

### Endpoints

The primary endpoints of this study were overall survival (OS) and relapse-free survival (RFS). Overall survival was defined as time until death from any cause; RFS was defined as time until relapse of disease or death from any cause. Secondary endpoints included non-relapse mortality (NRM), relapse incidence, and acute GVHD (grades II-IV), chronic GVHD (cGVHD), and times to neutrophil and platelet engraftment. NRM was defined as any cause of death occurring after HCT without documented relapse. Disease relapse was defined as relapse with or without death. Neutrophil engraftment was defined as the first day of an absolute neutrophil count > 500 cells/uL on 3 consecutive measurements. Platelet engraftment was defined as the first day of 2 consecutive measurements of >20,000 cells/uL and transfusion independence in the prior 7 days. Acute GVHD was graded based on MAGIC criteria, and chronic GVHD was graded according to Sullivan et al. [25, 26].

### HLA genotyping

High resolution genotyping of patients and donors was performed using NGS (ScisGo v4 and 5, Scisco Genetics, Seattle, WA) for HLA-A, B, C, DRB1/3/4/5, DQB1, DQA1, DPB1 and DPA1. ScisGo v4 kits were used prior to April 2017, and v5 kits were used after. NGS analysis was performed with GEMS software version 65.20 prior to Feb 2019, and version 97 after Feb 2019. In some cases, G group ambiguities remained after typing, particularly for HLA-DPB1. In these cases, the most common allele or allele combination was assigned based on CIWD version 3 [27].

### Mismatch determination and CHIME interaction

Allele-level mismatch was calculated for HLA-A, B, C, DRB1, DQB1, and DPB1 loci. EpMM was calculated by version 2 of the Eplet Mismatch program (Excel worksheets), which were previously available at http://www.epitopes.net/downloads.html. HLA-DRB1/3/4/5 EpMM are summed for the DR locus total EpMM; HLA-DQA1 and HLA-DPA1 EpMM are included in the HLA-DQ and HLA-DP totals, respectively. Both antibody-confirmed and predicted eplets were included in the EpMM total. Alleles unlisted in the worksheet were reassigned to the closest listed allele by known peptide sequence (i.e., the fewest eplet differences), and substitutions are listed in Supplementary Table 1. Some eplets were called inconsistently, possibly due to an unknown sequence at the time the worksheet was created; the v2 worksheets were corrected for alleles found in our cohort as described in Supplementary Table 2. Eplet mismatches were analyzed as continuous variables and at median cutoffs at each locus, class, and for host-vs-graft (HvG) and graft-vs-host (GvH) mismatch vectors. Differences exist in the eplet calls between HLA-matchmaker Excel worksheet versions and HLA Fusion matchmaker modules [28]. Alternative methods of eplet calculation are described in the Supplementary Methods.

EpMM divided at the median into high and low categories for each HLA class was used to create 4 interaction categories: 1. both classes low, 2. Class I low/Class II high, 3. Class I high/Class II low, and 4. Both classes are high. This interaction was first tested on HvG and GvH vectors independently, in which categories 1 and 4 were identified as higher risk for overall survival. Subsequently, HLA class EpMM were regrouped by HvG and GvH vector. This second interaction resulted in 16 groups: Categories 1-4 in HvG vector compounded by Categories 1-4 in GvH vector. The CHIME score was defined by the number of risk categories (1 or 4) present in the HvG and GvH vectors, with possible values of 0, 1, or 2.

### Statistical methods

Endpoints of OS and RFS are analyzed by Cox proportional hazards models. No violation of proportionality was found for the main predictors. Other endpoints entail competing risks (e.g., death before the end of the risk window) and are assessed by Fine and Gray modeling. Kaplan-Meier curves were compared with the log-rank test, and cumulative incidence curves were compared with Gray’s test. Post-hoc power calculation for overall survival was made using PASS software: 265 patients and 93 observed events (deaths) had 91.5% power to detect an HR of 2.0 when comparing low risk (50%) and high risk groups (50%) using a 2-sided, 0.05 significance level log-rank test.

All endpoints were assessed for bivariate association to HLA mismatch models and patient, donor, and transplant characteristics by Cox proportional hazards or Fine and Gray methods; initial multivariable models included predictors with *p* < 0.1. For inherently correlated variables (e.g., donor sex and female-to-male transplant), or categorical vs. continuous predictors, the lower *p*-value was included.

A stepwise method was used for each outcome variable to select baseline variables (see Supplementary Table 3) for the multivariable regression models. Variables with *p*-values ≤ 0.1 were retained in the final models.

*P* values for CHIME risk, derived from log-likelihood ratio tests within the multivariable regression models, were corrected for multiple testing across the ten outcomes using the Benjamini-Hochberg (BH) procedure to control the False Discovery Rate (FDR) [29]. Both raw and FDR-adjusted *p*-values are reported.

R software version is 3.6.3 (R Core Team, Vienna, Austria) with *cmprsk* v2.2-10*, survminer* v0.4.9 and *survival* v3.2-11 packages; all tests were 2-sided. PASS software was version 14.

## Results

### Patient and HSCT characteristics

The median age was 48 years (range: 8–76). Patient ethnicity and race were 52.8% Hispanic, 22.6% non-Hispanic White, 9.1% non-Hispanic Black, 14% Asian, and 1.5% Native Hawaiian/Pacific Islander. Diagnoses included AML (38.5%), ALL (28.3%), MDS/MPN (18.5%), and lymphoma (12.1%). High/very high DRI was present in 34.7%, HCT-CI > 2 in 44.9%, and 89.4% had KPS ≥ 80. Donor median age was 34 years; 94% received PBSC graft. Physical (flow cytometric) crossmatch results were available for 260 patients. Antibody tests for patients with positive or missing crossmatch were reviewed for donor-specific antibodies (DSA). Eight patients had intermediate/strong DSA (higher than 3000 MFI), of whom seven underwent desensitization. Conditioning was myeloablative in 49.4% and RIC/NMA in 50.6%. Most received PTCy/MMF with tacrolimus (98.1%), with sirolimus as an alternative for tacrolimus. The distribution of specific HLA allele mismatches is included in Table 1.

**Table 1:** Patient and Transplant Characteristics with Categories of Interest.

	Cohort (*n* = 265)	CHIME 0 (*n* = 64)	CHIME 1 (*n* = 134)	CHIME 2 (*n* = 67)	p*
**Patient characteristics**					
**Patient Age at HCT, years**					
Median	48	46	46	54	0.19
Interquartile range	31, 62	31, 59	32, 62	37, 66	
Range	(8–76)	(8–76)	(9–76)	(12–75)	
**Patient sex**					
Female	98 (37%)	27 (42.2%)	46 (34.3%)	25 (37.3%)	0.56
**Patient race**					
Hispanic	140 (52.8%)	31 (48.4%)	71 (53%)	38 (56.7%)	0.33
White (non-Hispanic)	60 (22.6%)	12 (18.8%)	32 (23.9%)	16 (23.9%)	
Asian	37 (14%)	14 (21.9%)	16 (11.9%)	7 (10.4%)	
Black (non-Hispanic)	24 (9.1%)	7 (10.9%)	11 (8.2%)	6 (9%)	
Native Hawaiian/Pacific Islander	4 (1.5%)	0 (0%)	4 (3%)	0 (0%)	
**Primary diagnosis HCT**					
AML	102 (38.5%)	26 (40.6%)	52 (38.8%)	24 (35.8%)	0.65
ALL	75 (28.3%)	16 (25%)	43 (32.1%)	16 (23.9%)	
MDS/CML/MPN	49 (18.5%)	10 (15.6%)	22 (16.4%)	17 (25.4%)	
Lymphoma	32 (12.1%)	9 (14.1%)	14 (10.4%)	9 (13.4%)	
Multiple Myeloma/ Other malignancy	7 (2.6%)	3 (4.7%)	3 (2.2%)	1 (1.5%)	
**DRI score**					
Low	53 (20%)	17 (26.6%)	23 (17.2%)	13 (19.4%)	0.36
Intermediate	120 (45.3%)	25 (39.1%)	65 (48.5%)	30 (44.8%)	
High	82 (30.9%)	22 (34.4%)	40 (29.9%)	20 (29.9%)	
Very high	10 (3.8%)	0 (0%)	6 (4.5%)	4 (6%)	
**Karnofsky performance status %**					
80–100	237 (89.4%)	59 (92.2%)	120 (89.6%)	58 (86.6%)	0.55
<80	27 (10.2%)	5 (7.8%)	13 (9.7%)	9 (13.4%)	
missing*	1 (0.4%)	0	1 (0.7%)	0	
**HCT comorbidity index**					
0	63 (23.8%)	19 (29.7%)	34 (25.4%)	10 (14.9%)	0.33
1–2	81 (30.6%)	18 (28.1%)	43 (32.1%)	20 (29.9%)	
>2	119 (44.9%)	27 (42.2%)	57 (42.5%)	35 (52.2%)	
missing*	2 (0.8%)	0	0	2 (3%)	
**Donor characteristics**					
**Donor age**					
Median	34	32	36	34	0.66
Interquartile range	25, 43	24, 43	25, 43	26–41	
Range	(11–68)	(13–60)	(11–68)	(12–60)	
**Donor sex**					
Female	92 (34.7%)	25 (39.1%)	46 (34.3%)	21 (31.3%)	0.64
**Relationship to patient**					
Parent	25 (9.4%)	5 (7.8%)	14 (10.4%)	6 (9%)	0.5
Child	124 (46.8%)	26 (40.6%)	60 (44.8%)	38 (56.7%)	
Sibling	96 (36.2%)	26 (40.6%)	50 (37.3%)	20 (29.9%)	
Other (second-degree)	20 (7.5%)	7 (10.9%)	10 (7.5%)	3 (4.5%)	
**Transplant characteristics**					
**Stem cell source**					
Bone Marrow	16 (6%)	6 (9.4%)	5 (3.7%)	5 (7.5%)	0.25
Peripheral Stem Cells	249 (94%)	58 (90.6%)	129 (96.3%)	62 (92.5%)	
**Graft CD34+ dose (target dose 5 x 10**^**6**^**/kg)**					
< 4.5 x 10^6^/kg	71 (26.8%)	23 (35.9%)	33 (24.6%)	15 (22.4%)	0.15
4.5 to 5.5 x 10^6^/kg	157 (59.2%)	33 (51.6%)	78 (58.2%)	46 (68.7%)	
> 5.5 x 10^6^/kg	28 (10.6%)	6 (9.4%)	18 (13.4%)	4 (6%)	
missing*	9 (3.4%)	2 (3.1%)	5 (3.7%)	2 (3.0%)	
**Graft CD3+ dose (median 1.77 ×10**^**8**^**/kg)**					
>median	137 (51.7%)	35 (54.7%)	66 (49.3%)	36 (53.7%)	0.71
≤median	118 (44.5%)	27 (42.2%)	63 (47%)	28 (41.8%)	
missing*	10 (3.8%)	2 (3.1%)	5 (3.7%)	3 (4.5%)	
**Female donor to male patient**					
Yes	50 (18.9%)	13 (20.3%)	25 (18.7%)	12 (17.9%)	0.94
No	215 (81.1%)	51 (79.7%)	109 (81.3%)	55 (82.1%)	
**ABO blood group compatibility**					
ABO compatible	188 (70.9%)	45 (70.3%)	95 (70.9%)	48 (71.6%)	0.4
Minor mismatch (donor is O)	28 (10.6%)	10 (15.6%)	10 (7.5%)	8 (11.9%)	
Major mismatch (recipient is O)	29 (10.9%)	7 (10.9%)	17 (12.7%)	5 (7.5%)	
Bidirectional (none are O)	20 (7.5%)	2 (3.1%)	12 (9%)	6 (9%)	
**Donor/recipient CMV serostatus**					
D-/R-	20 (7.5%)	7 (10.9%)	5 (3.7%)	8 (11.9%)	0.3
D-/R+	59 (22.3%)	15 (23.4%)	29 (21.6%)	15 (22.4%)	
D + /R-	25 (9.4%)	4 (6.3%)	12 (9%)	9 (13.4%)	
D + /R+	159 (60%)	37 (57.8%)	87 (64.9%)	35 (52.2%)	
missing*	2 (0.8%)	1 (1.6%)	1 (0.7%)	0	
**Conditioning regimen**					
Fludarabine/Melphalan/TBI	86 (32.5%)	18 (28.1%)	45 (33.6%)	23 (34.3%)	0.59
Fludarabine/Cy/TBI	35 (13.2%)	9 (14.1%)	14 (10.4%)	12 (17.9%)	
Fludarabine/FTBI	77 (29.1%)	19 (29.7%)	42 (31.3%)	16 (23.9%)	
Fludarabine/Cy/TMLI	31 (11.7%)	6 (9.4%)	15 (11.2%)	10 (14.9%)	
Other^†^	36 (13.6%)	12 (18.8%)	18 (13.4%)	6 (9%)	
**Conditioning regimen intensity**					
Ablative	131 (49.4%)	35 (54.7%)	70 (52.2%)	26 (38.8%)	0.13
Non-myeloablative/Reduced Intensity	134 (50.6%)	29 (45.3%)	64 (47.8%)	41 (61.2%)	
**GVHD prophylaxis**					
Tacrolimus/Cy/MMF	260 (98.1%)	63 (98.4%)	132 (98.5%)	65 (97%)	0.84
Sirolimus/Cy/MMF	5 (1.9%)	1 (1.6%)	2 (1.5%)	2 (3%)	
**Year of HCT**					
2011–2016	61 (23%)	17 (26.6%)	34 (25.4%)	10 (14.9%)	0.19
2017–2020	204 (77%)	47 (73.4%)	100 (74.6%)	57 (85.1%)	
**Allele mismatch**					
Overall					
6/12 matched	164 (61.9%)	37 (57.8%)	86 (64.2%)	41 (61.2%)	0.08
7/12 matched	73 (27.5%)	23 (35.9%)	34 (25.4%)	16 (23.9%)	
8/12 matched	21 (7.9%)	4 (6.3%)	12 (9%)	5 (7.5%)	
9+ matched	7 (2.6%)	0 (0%)	2 (1.5%)	5 (7.5%)	
Class I					
3/6 matched	225 (84.9%)	56 (87.5%)	115 (85.8%)	54 (80.6%)	0.78
4/6 matched	33 (12.5%)	6 (9.4%)	16 (11.9%)	11 (16.4%)	
5/6 or 6/6 matched	7 (2.6%)	2 (3.1%)	3 (2.2%)	2 (3%)	
Class II (HLA-DRB1, DQB1, DPB1)					
3/6 matched	190 (71.7%)	44 (68.8%)	101 (75.4%)	45 (67.2%)	0.25
4/6 matched	61 (23%)	19 (29.7%)	25 (18.7%)	17 (25.4%)	
5/6 or 6/6 matched	14 (5.3%)	1 (1.6%)	8 (6%)	5 (7.5%)	
Individual Locus					
HLA-A	250 (94.3%)	63 (98.4%)	125 (93.3%)	62 (92.5%)	0.25
HLA-B	253 (95.5%)	60 (93.8%)	130 (97%)	63 (94%)	0.44
B-leader or allele match	156 (58.9%)	44 (68.8%)	74 (55.2%)	38 (56.7%)	0.19
B-leader unknown (rare allele)	1 (0.4%)	0	1 (0.7%)	0	
HLA-C	244 (92.1%)	59 (92.2%)	124 (92.5%)	61 (91%)	0.95
HLA-DRB1	254 (95.8%)	63 (98.4%)	127 (94.8%)	64 (95.5%)	0.56
HLA-DQB1	234 (88.3%)	56 (87.5%)	119 (88.8%)	59 (88.1%)	0.97
HLA-DPB1	217 (81.9%)	52 (81.3%)	115 (85.8%)	50 (74.6%)	0.15
Permissive	153 (57.7%)	40 (62.5%)	79 (59%)	34 (50.7%)	0.43
HvG non-permissive	34 (12.8%)	8 (12.5%)	20 (14.9%)	6 (9%)	
GvH non-permissive	30 (11.3%)	4 (6.3%)	16 (11.9%)	10 (14.9%)	
Desensitization or Donor-specific Antibody	8 (3.0%)	1 (1.6%)	5 (3.7%)	2 (3.0%)	0.89

*Unknown/missing categories were excluded from the chi-square calculation.

^†^Other regimens include Busulfan with or without Fludarabine and TBI (*n* = 10), FTBI/FLU/Cy (*n* = 5), TMLI/FLU with or without MELPHALAN (*n* = 15), FLU/TBI only (*n* = 5), and FLU/Cy/TBI with anti-thymocyte globulin(*n* = 1).

*ALL* Acute Lymphocytic Leukemia, *AML* Acute Myeloid Leukemia, *Cy* cyclophosphamide, *DRI* Disease Risk Index, *FTBI* Fractionated Total Body Irradiation, *GvH* Graft-vs.Host *HvG* Host-vs.-Graft, *MDS/CML/MPN* Myelodysplastic Syndrome/Chronic Myelomonocytic Leukemia/Myeloproliferative Neoplasms, *MMF* mycophenolate mofetil, *NA* not applicable, *TBI* Total Body Irradiation, *TMLI* Total marrow and lymphoid irradiation.

### Eplet-derived epitope mismatch

Higher EpMM in GvH direction correlated with lower EpMM in HvG direction for each HLA class (class I, R = –0.15 *p* = 0.012; class II, R = –0.21, *p* < 0.001, Supplementary Fig. 1). The median EpMM for HLA class I was 10 (range, 0–27), class II was 24 (range 0–62), and the median total EpMM was 34 (range 0–82). Eplet mismatch level by class and mismatch vector did not differ significantly between ethnic groups, except for the Native Hawaiian/Pacific Islander group having significantly less GvH HLA Class I EpMM (*n* = 4, see Table 1; Supplementary Table 4)

Eplet mismatch for individual loci was correlated with outcomes in two instances. Median follow-up for surviving patients was 24.5 months (range, 3.3 to 75.7). GvH vector HLA-A EpMM, when modeled as a continuous variable, associated with increased survival (*p* = 0.024) and RFS (*p* = 0.006). HLA-B HvG EpMM above the median associated with reduced incidence of cGvHD (*p* = 0.037). However, neither was significant after adjustment for the demographic factors identified in Supplementary Table 3.

For total eplet mismatch within class I and II, HvG class II EpMM above median associated with increased aGvHD grade II–IV (HR 1.50, 95% CI 1.02–2.2, *p* = 0.040 after MVA). GvH class II and total (i.e., class I + class II) eplet mismatch higher than the median associated with longer time to platelet engraftment, with total EpMM being the stronger predictor (HR 1.49, 95% CI 1.15– 1.94, *p* = 0.003 after MVA). However, a similar and stronger effect on platelet engraftment was observed for DRB1 allele-level mismatch in the GvH direction (HR 3.53 (2.10–5.46), *p* < 0.001), indicating that allele-level mismatch was more predictive than EpMM for platelet engraftment.

### EpMM class interaction and CHIME

When EpMM was dichotomized at median by HLA class and mismatch vector (HvG, GVH), patients with high EpMM in only one class had improved survival (*p* = 0.029 GvH, *p* = 0.014 HvG) and RFS (*p* = 0.11 GvH, *p* = 0.010 HvG) compared to individuals with low or high EpMM in both classes (Supplementary Fig. 2). Survival differences were driven by NRM, not relapse (Supplementary Fig. 3). Class I/II interactions of EpMM were associated with aGvHD II-IV in the HvG direction (*p* = 0.023) but not severe aGVHD (grade III-IV), cGvHD or engraftment (Supplementary Fig. 4).

**Fig. 1:**
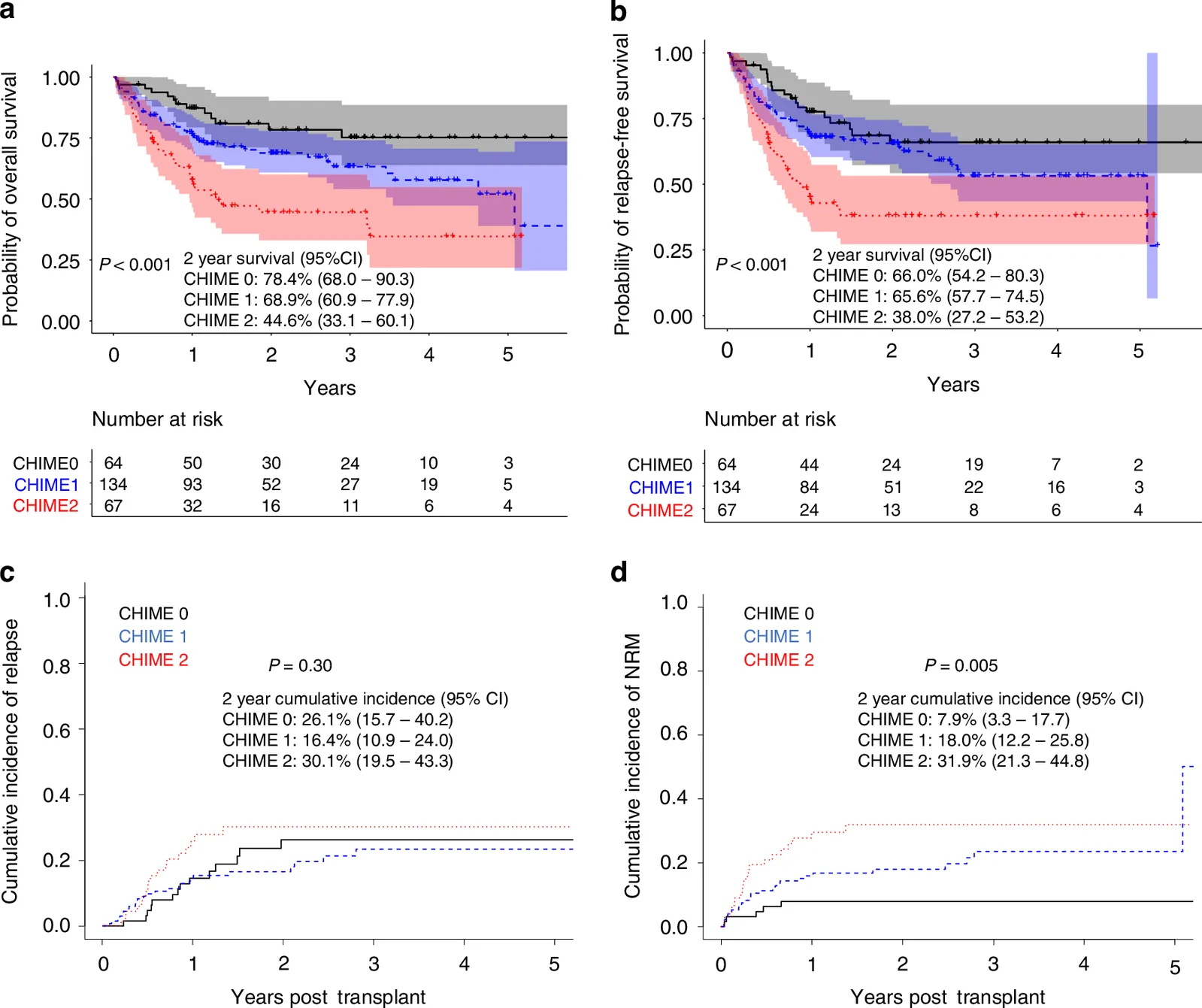
CHIME risk groups, survival, relapse, and NRM. **a** Probability of overall survival, **b** relapse-free survival; **c** cumulative incidence of relapse, and **d** non-relapse mortality.

**Fig. 2:**
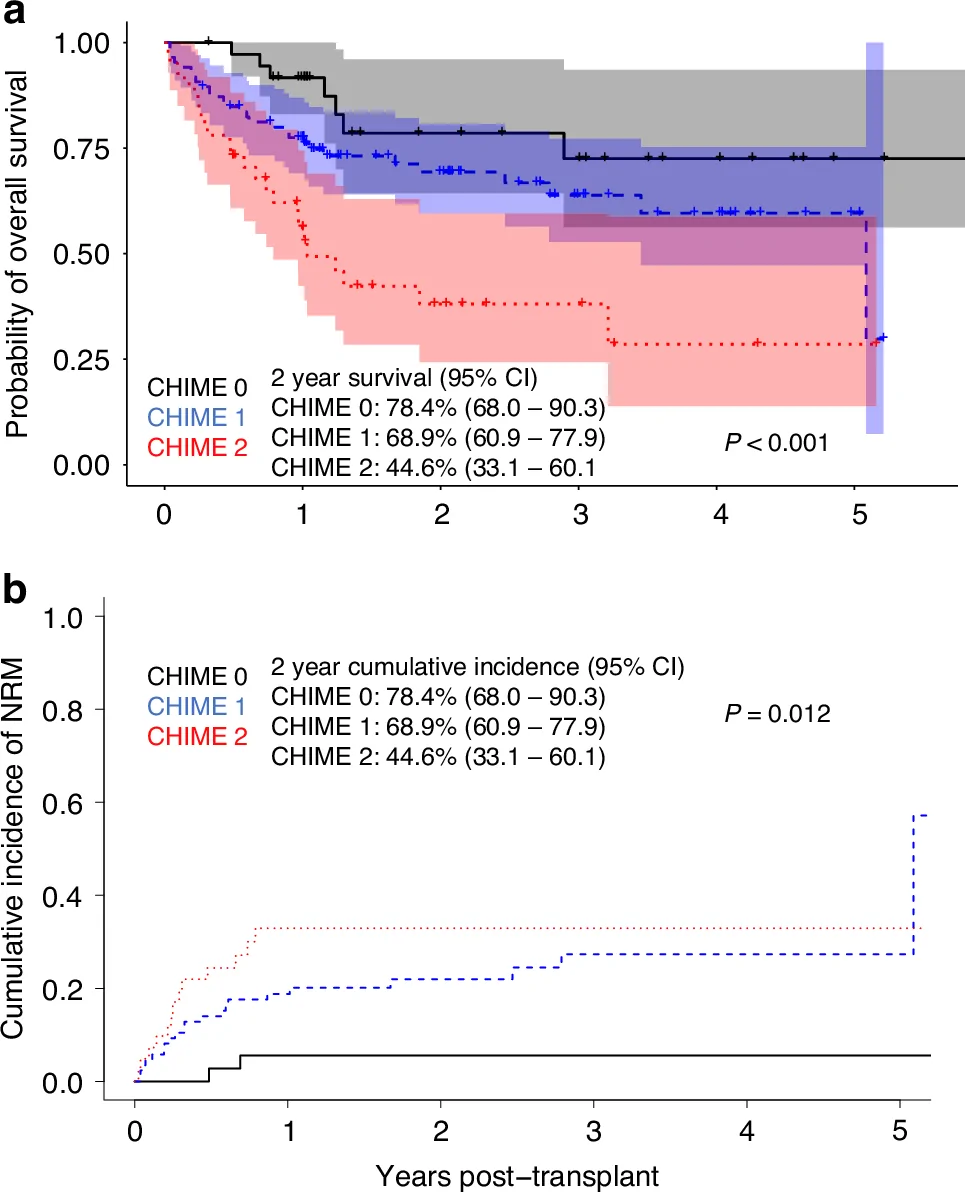
Survival and NRM by the CHIME group in 6/12 mismatched patients. **a** Overall survival, **b** NRM.

From the two vectors’ interactions, a second-degree interaction was modeled (i.e., CHIME, see Table 2). CHIME model significantly associated with overall survival (*p* < 0.001), RFS (*p* < 0.001), and NRM (*p* = 0.005), but not relapse (*p* = 0.30) (Fig. 1A–D). The CHIME score did not significantly associate with the incidence of acute or chronic GvHD (Supplementary Fig. 5) or time to engraftment (not shown). To exclude effects of allele-level matching, we repeated univariate analyses in the 6/12 mismatch subset (*N* = 164); CHIME remained significantly associated with OS (*p* < 0.001) and NRM (*p* = 0.012) (Fig. 2). In multivariable analysis, CHIME groups independently predicted OS, RFS, NRM and severe grade III-IV aGVHD (Table 3).

**Table 2:**
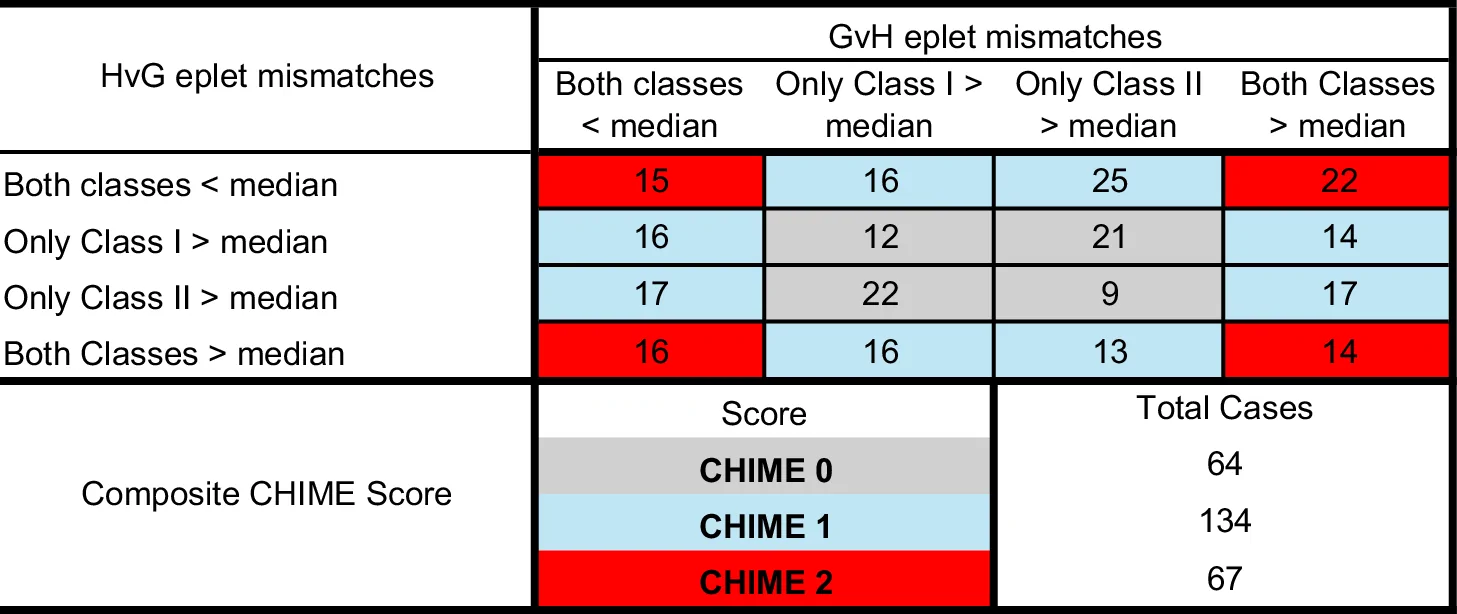
Bidirectional eplet mismatch class interaction groups.

Chi-square *p*-value = 0.22. Eplet mismatch (EpMM) imbalance is defined as > median EpMM in both HLA classes or ≤ median in both classes in a single mismatch vector. Interaction groups with imbalance in both vectors are highlighted red (i.e, CHIME 2); one vector imbalance is highlighted blue (i.e., CHIME 1), and neither vector imbalance is highlighted gray (i.e., CHIME 0)

**Table 3:**
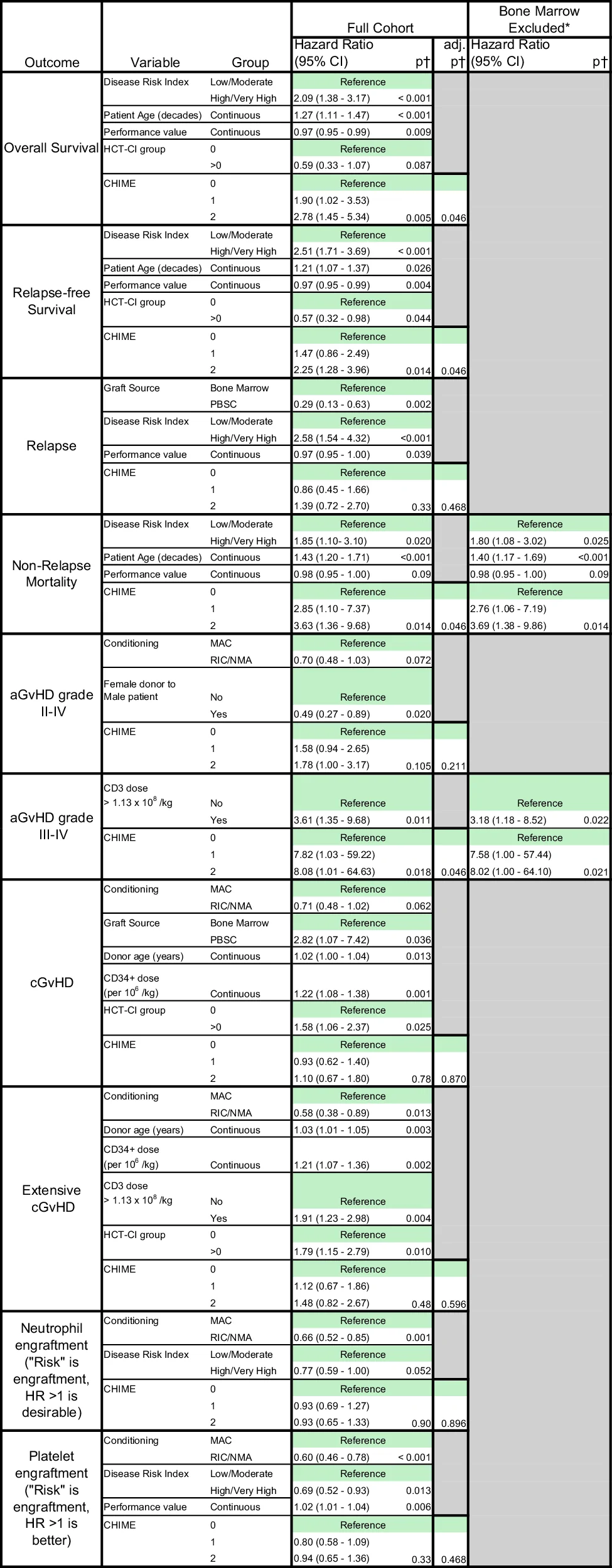
Multivariate analysis of outcomes with CHIME and significant univariate demographic variables.

* There were no instances of Non-Relapse Mortality or aGvHD grade III or IV in the 16 patients with bone marrow stem cell source; these outcomes were analyzed without the stem cell source variable (left), and with bone marrow subset excluded (right).

p†: raw *p* value for model predictors.

adj. p† : CHIME predictor Benjamini-Hochberg method corrected p for full cohort.

We analyzed EpMM and CHIME using the 2017 and 2020 EpMM libraries in HLA Fusion software v4.6.1 (2017 median Class I 26, class II 25; 2020 median Class I 19, class II 26). Reassigned CHIME scores showed weaker associations with OS and were not further analyzed (Supplementary Fig. 6).

### CHIME effects show interactions with CD3 T cell dose

**Fig. 3:**
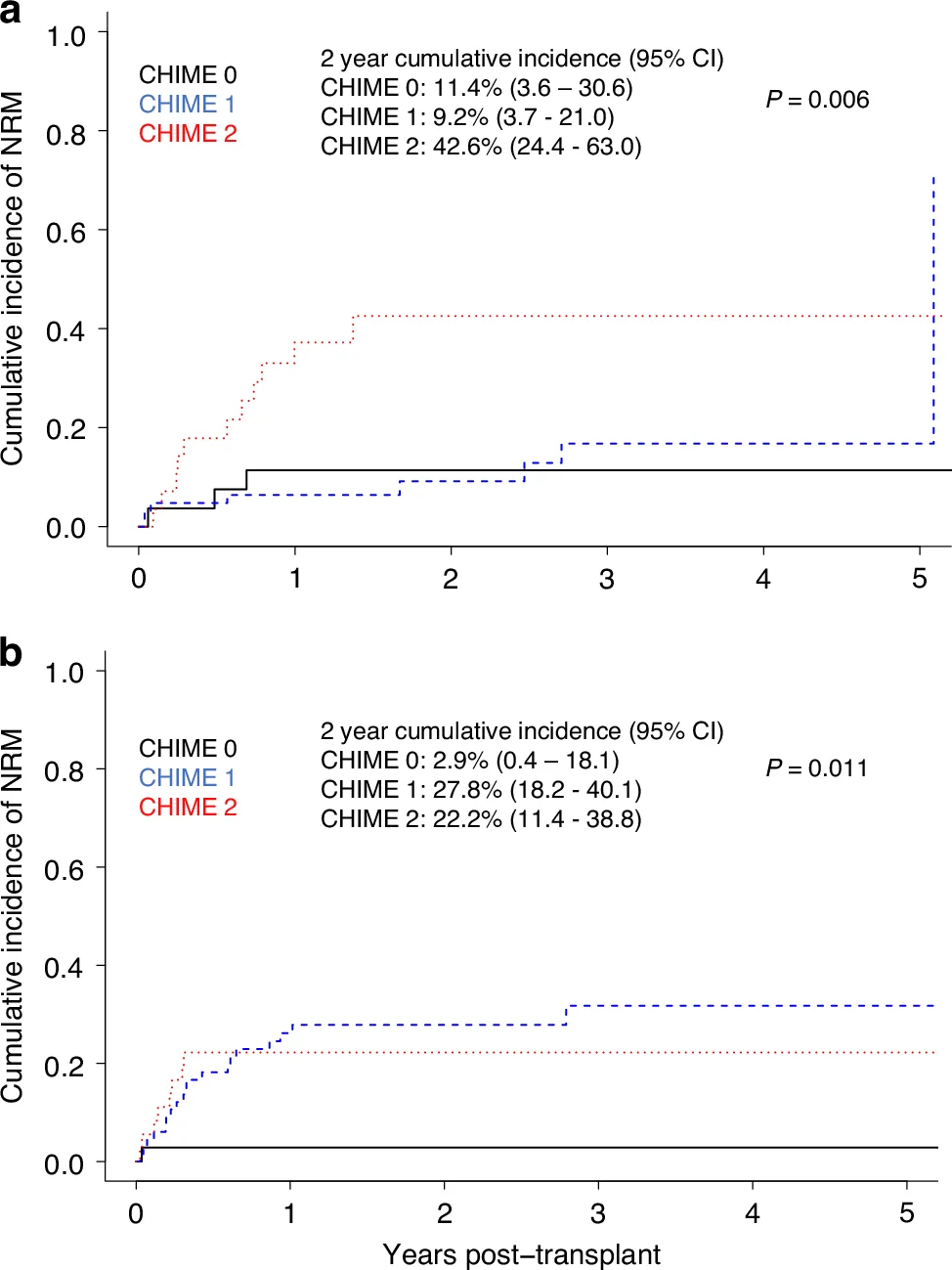
CHIME group and NRM cumulative incidence stratified by CD3 dose. **a** CD3 graft dose ≤ 1.77 × 10^8^/kg, **b** CD3 graft dose > 1.77 ×10^8^/kg.

Because graft CD3+ cell dose > 1.77 × 10^8^ cells/kg was associated with GvHD in univariate analysis (Supplementary Table 3), we stratified CHIME by CD3 dose. CHIME 0 was protective and CHIME 2 conferred higher NRM in both strata, while CHIME 1 was higher risk only with high CD3 (Fig. 3). Grouping CHIME 0 and CHIME 1/low CD3 as low risk, and CHIME 1/high CD3 and CHIME 2 as high risk, the high-risk group was associated with severe CRS (grade 3–4) (Table 4, *p* = 0.011). Although HLA-DRB1 allele mismatch was linked to CRS ≥ grade 1 (*p* = 0.050), the CHIME-CD3 association persisted after excluding DRB1-matched cases (*N* = 37, *p* = 0.03). The high-risk group had greater GvHD-related (OR 6.16, CI 1.33–57.8, *p* = 0.011) and organ dysfunction-related mortality (OR 5.05, CI 1.05–48.4, *p* = 0.035), with similar relapse-related death between groups (Supplementary Table 5).

**Table 4:** Incidence of CRS by CHIME group and CD3 dose.

Outcome	CHIME 0, or CHIME 1 with low CD3	CHIME 2, or CHIME 1 with high CD3	OR (95% CI)	*p*
CRS (Full Cohort*)				
CRS 0-1	89	76	0.57 (0.33–0.98)	0.039
CRS 2	36	45	1.29 (0.74– 2.27)	0.35
CRS 3-4	2	12	6.16 (1.33– 57.8)	0.011
CRS (excluding GvH and/or HvG matched DRB1 allele)				
CRS 0-1	74	65	0.66 (0.37–1.18)	0.17
CRS 2	34	37	1.08 (0.60–1.99)	0.78
CRS 3-4	2	11	5.78 (1.22– 54.9)	0.019

*n = 260; Graft CD3 data for 5 patients in CHIME 1 category were missing.

## Discussion

The expanding use of HaploHCT with PTCy has increased access to curative therapy for patients without suitable matched donors. Optimal donor selection remains under study, particularly the impact of HLA disparity in the non-shared haplotype. Beyond antigen- and allele-level mismatching, eplet-based models have emerged. In our analysis, we developed a novel model of eplet mismatch interaction between HLA classes, CHIME, that associates with improved overall survival and NRM.

Although multiple molecular models exist for quantifying HLA mismatch, we focused on eplet mismatch due to several considerations. PIRCHE models predict indirect alloreactivity [30], but not direct alloreactivity or differences in peptide repertoires presented by mismatched antigens. EMMA [14], a model similar to eplet mismatch, lacks functional stratification aside from solvent exposure. In contrast, eplets represent potential binding targets enriched in residues near the peptide-binding groove. Although initially developed to predict B-cell receptor reactivity for solid organ transplantation [16], eplet mismatch potentially bridges gaps in alloreactive prediction of other models. Comprising one or more peptide mismatches, each eplet represents a possible increase in PIRCHE and EMMA scores. Eplets near and within the T-cell receptor binding site could also contribute to direct allorecognition, or by changing critical anchor residue preferences in the binding site, drastically alter the immunopeptidome between patient and donor. By representing a mixture of possible targeting models, eplets provide a biologically relevant framework for assessing alloimmune risk.

We initially found only a limited association between eplet mismatch number and patient outcomes, which agrees with a larger multi-center analysis by Zou and colleagues [21]. Class II HvG EpMM association with aGvHD was marginal and not corrected for multiple testing. Notably, increased class II and total eplet mismatch in the GvH direction was associated with delayed platelet engraftment, a finding consistent with prior CIBMTR data but potentially reflective of the retrospective nature of the study rather than a biological effect. This association is confounded by the finding that HLA-DRB1 allele-level mismatch in the GvH direction is a stronger predictor of delayed engraftment than eplet mismatch. The absence of DSA data in the CIBMTR cohort may have contributed to this finding, whereas few patients in our cohort had clinically significant DSAs prior to HaploHCT. We subsequently modeled CHIME as an interaction in eplet mismatch between HLA class and mismatch vector, with significant effects observed for OS, RFS and NRM, but not relapse. The CHIME model suggests that the effect of molecular mismatch is not simply additive across HLA class and mismatch vector. Within HaploHCT, an optimal balance of class I and class II mismatches in the non-shared haplotype may enhance graft-versus-leukemia (GvL) effects while mitigating GvHD risk and NRM.

One potential mechanism for linking EpMM to NRM is CRS, which is common after HaploHCT due to rapid alloreactive T-cell expansion and the associated elevated circulating levels of pro-inflammatory cytokines [31–35]. Although the impact of low-grade CRS is unclear [31, 33–36], higher graft CD3+ cell dose and HLA-DRB1 or non-permissive HLA-DPB1 mismatch have been associated with CRS [33, 35, 37, 38]. We previously demonstrated that graft CD3 cell dose and HLA mismatch in HaploHCT were predictive of severe CRS, which was associated with higher NRM [35]. In this study, CHIME interacted with graft CD3+ dose in relation to NRM. While NRM was consistently low in CHIME 0 and high in CHIME 2, CHIME 1 patients had increased severe CRS and NRM only with high CD3+ grafts. Even low-grade CRS has been linked to infectious complications [34, 38–40], and earlier fever onset may predict higher mortality [41, 42]. These findings suggest that integrating HLA mismatch models with graft composition may refine risk stratification in HaploHCT, though validation in larger cohorts is needed.

Our study is limited by its single-center, retrospective design and modest sample size. Low allele-match rates at most loci, including DPB1 T-cell epitope non-permissive mismatch, reduced power for allele-level analyses, and several observed statistical associations were marginal. With the limited cohort size and exploratory nature, we did not optimize cutoffs.

EpMM/CHIME performance may also vary by calculation approach and software version [28]. We used corrected v2 worksheets (as in Rimando et al.), whereas Zou used Matchmaker software, which likely contributed to differences in median EpMM. We acknowledge that v2 worksheets may no longer be available at the original http://www.epitopes.net/downloads.html, please refer to the Data Availability statement for alternatives. Our median EpMM matches values reported by Rimando et al., while Class I is lower and Class II is higher than Zou et al.'s median values, due to differences in the calculation method of v2 worksheets vs. Fusion Matchmaker software and limited Class II genotyping in the CIBMTR data set. In our Supplementary results, we noted the eplet calculation method had a significant impact on the strength of observed associations (Supplementary methods and results, Supplementary Fig. 6). The variation of calculated EpMM by platform, version, and genotype data availability highlights the need for a consensus method of EpMM calculation, based on an externally validated database, for future studies. Lastly, the high median EpMM in HaploHCT may not apply to unrelated donor cases.

With further validation, this model could help refine donor selection strategies in HaploHCT with PTCy and serve as a prognostic tool for complications when alternative donors are not available. Future studies should also evaluate its applicability in mismatched unrelated donor HCT with PTCy, which is ongoing.

## Supplementary information

Supplementary Materials

## Data Availability

R core software and packages are publicly available at https://cran.r-project.org/. R script is available on request from the corresponding author.

## Data availability

The datasets generated and analyzed during the current study are available from the corresponding author on reasonable request. Authors were notified during review that the URL http://www.epitopes.net/downloads.html may no longer be accessible. An archived version of the page may be viewed at https://web.archive.org; the corresponding author can provide the original and corrected versions of the v2 worksheets on reasonable request.
