# Supplementary material for: CHIME: a novel HLA eplet model associates with non-relapse mortality in haploidentical hematopoietic cell transplant with post-transplant cyclophosphamide
Source: Bone Marrow Transplant. 2026 Jul 4;61(9):1228–37. doi: 10.1038/s41409-026-02954-6 (PMC13569419; doi:10.1038/s41409-026-02954-6)
Supplement: Supplementary file 1 [file 41409_2026_2954_MOESM1_ESM.pdf]

Supplemental Information

Supplemental Methods

Alternative EpMM calculation and v2 worksheet correction

Excel worksheet versions 3.1 (class II) and 4.0 (class I) from http://www.epitopes.net/downloads were also used for comparative calculation, as well as HLA Fusion Matchmaker analysis module libraries 2017 and 2020 (One Lambda, v4.4).

Most of the differences are due to eplets added, deleted, redefined or changed in terms of calculation (i.e., inter-locus eplet mismatch calculation) between versions. We did not attempt to reconcile eplet definitions between versions. Some variation was due to inconsistencies in the application of eplet definitions in version 2 worksheets. To correct these inconsistencies, we compared eplet mismatch determinations between versions 2, 3.1, and 4 worksheets, and at https://www.epregistry.com.br to identify inconsistent eplet calls that affected alleles within our data set, then recalculated the eplet mismatch to produce a corrected eplet mismatch value. The corrected eplet mismatch values are used for the analyses in the main text and figures. A list of substituted alleles for alleles not included in the v2 worksheet, and a list of corrections made to the v2 worksheet are found in Supplemental Tables 1 and 2.

Supplemental Results

CHIME calculation with other EpMM calculator versions

Calculation of class II eplet mismatch with version 3.1 worksheets resulted in a significantly reduced median eplet mismatch. Eplets calculated using the One Lambda HLA Fusion Matchmaker module yields significantly higher class I median EpMM, while median class II EpMM is similar to the version 2 worksheet.

CHIME models calculated with median cutoffs using the 3.1 and 4.0 worksheets were not significant for patient outcomes (not shown), while CHIME models with HLA Matchmaker software (HLA Fusion, One Lambda) were less statistically significant for patient survival (Supplemental Figure 6) than with v2 worksheets.

Supplemental Figure 1: EpMM correlation between GvH and HvG direction. (A) Class I EpMM correlation, (B) Class II EpMM correlation.

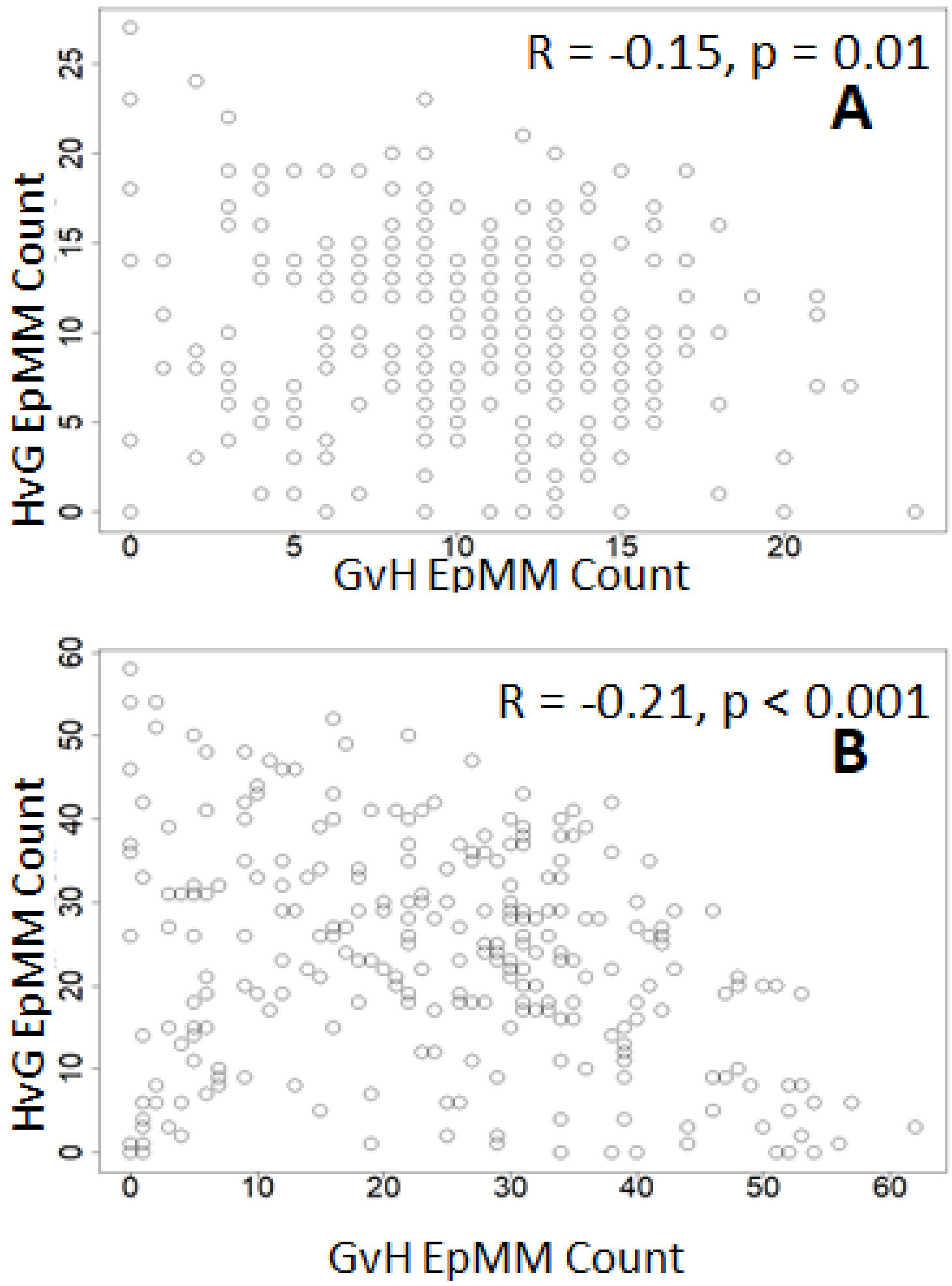

Supplemental Figure 2: Survival outcomes by eplet mismatch groups. Y-axis denotes probability of outcome, x-axis denotes years after transplant. (A) Overall survival by HLA class eplet mismatch interaction in HvG and (B) GvH direction. (C) Relapse-free survival by HvG and (D) GvH direction.

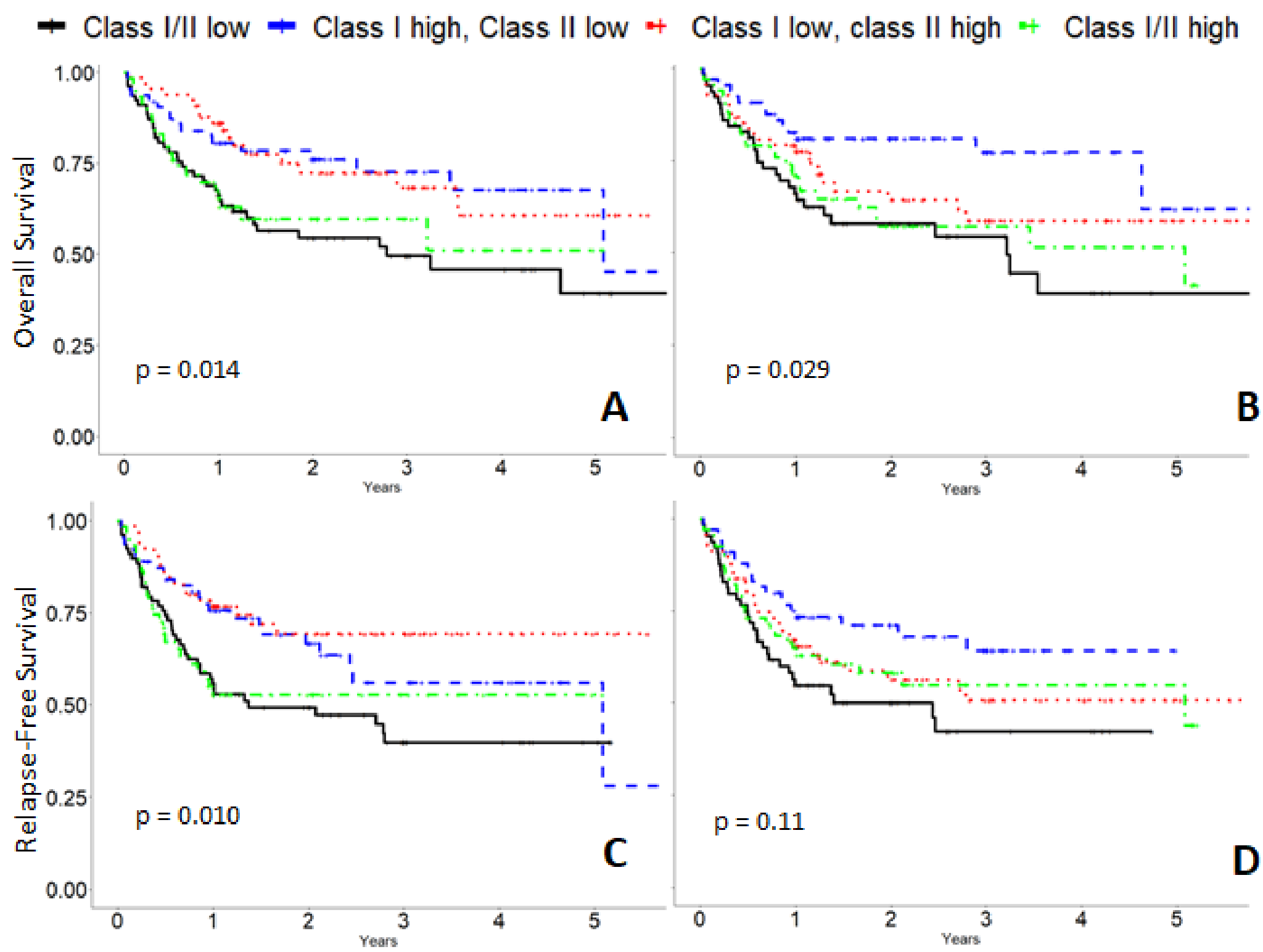

Supplemental Figure 3: Relapse and NRM by eplet mismatch groups. (A) Relapse incidence by HLA Class eplet mismatch interaction in HvG direction and (B) GvH direction. (C) Cumulative incidence of non-relapse mortality by HvG direction and (D) GvH direction.

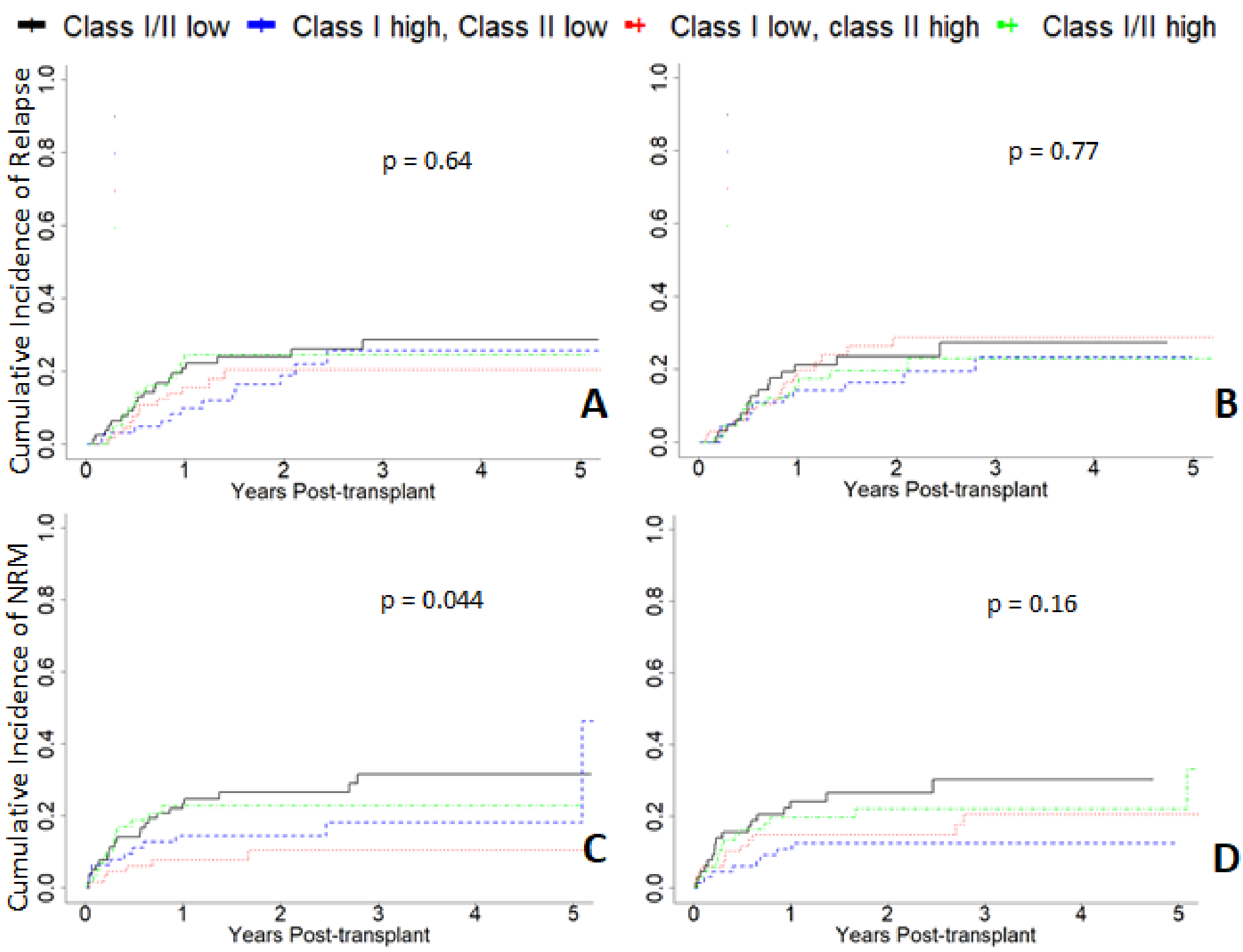

Supplemental Figure 4. Additional patient outcomes' cumulative incidence by eplet mismatch interaction and direction. (A) aGvHD grade II-IV by in HvG and (B) GvH direction. (C) aGvHD III-IV by HvG and (D) GvH direction. (E) cGvHD by HvG and (F) GvH direction. (G) Extensive cGvHD by HvG and (H) GvH direction. (I) Neutrophil engraftment by HvG and (J) GvH direction. (K) Platelet engraftment by HvG and (L) GvH direction.

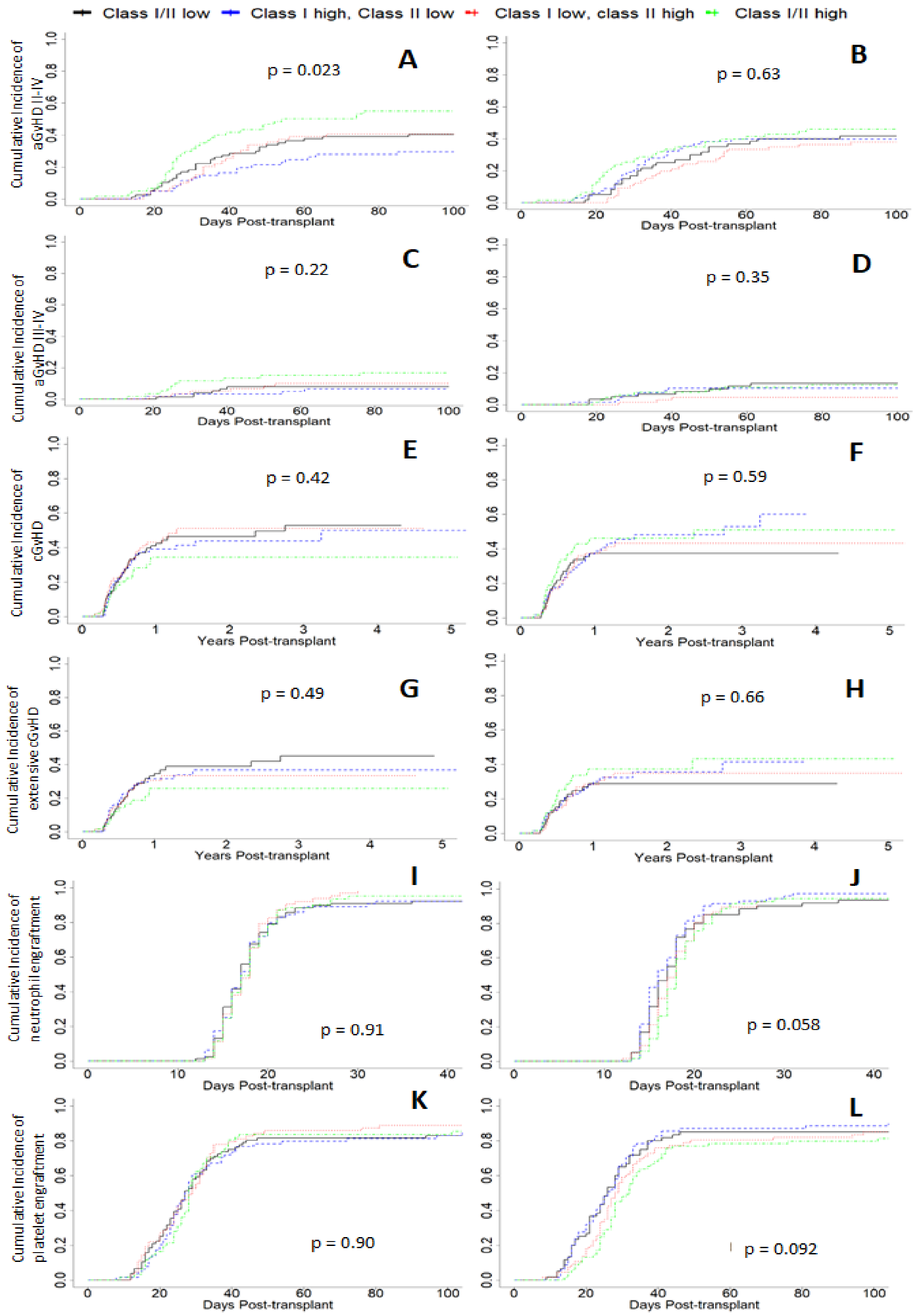

Supplemental Figure 5: CHIME groups and cumulative incidence of GvHD outcomes. (A) Any cGvHD, (B) extensive cGvHD, (C) aGvHD grade II-IV, (D) aGvHD grade III-IV.

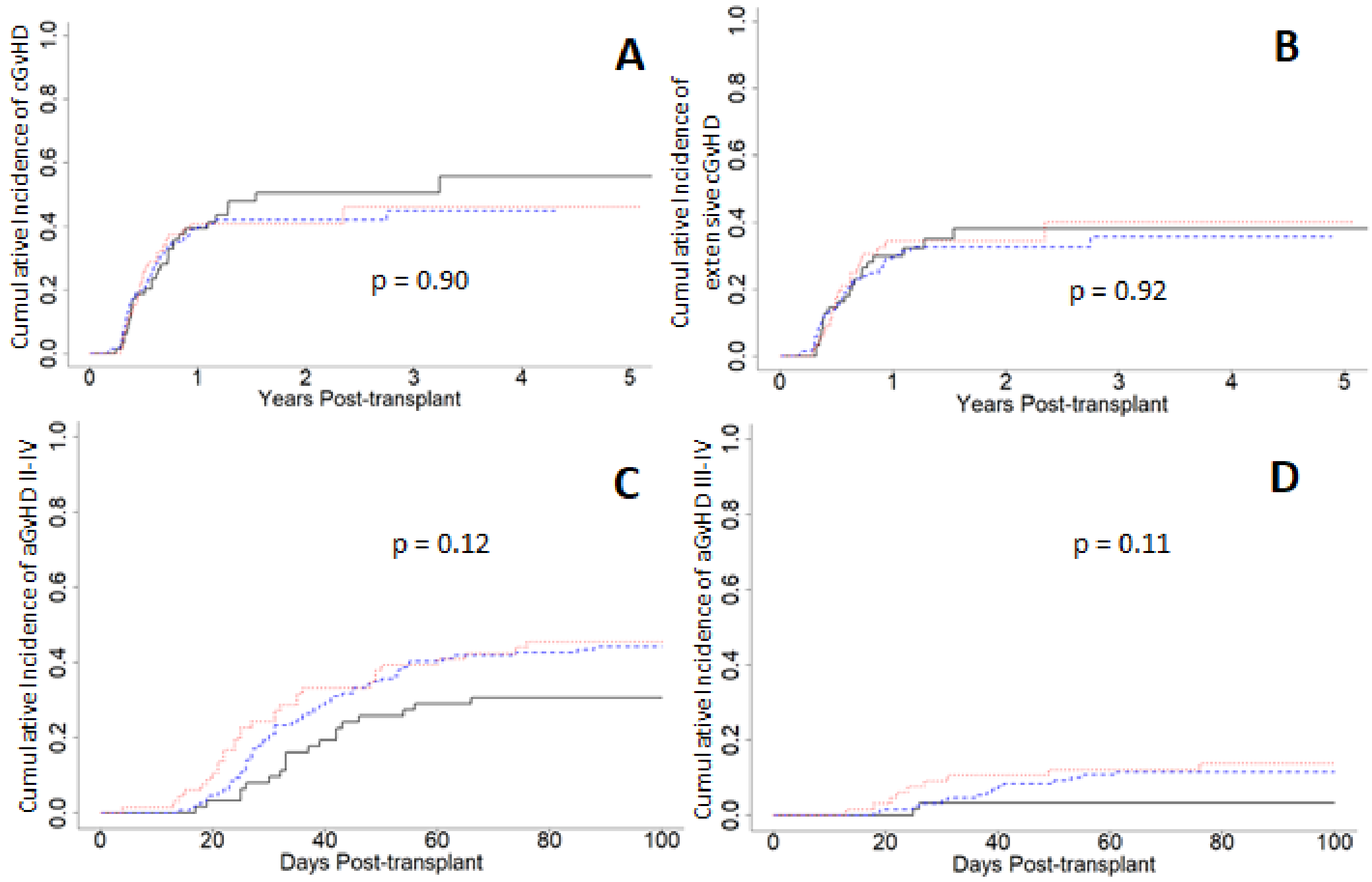

Supplemental Figure 6. Overall Survival and CHIME using Fusion Matchmaker software

A) CHIME calculated with 2017 eplet Fusion library. B) CHIME calculated with 2020 eplet Fusion library.

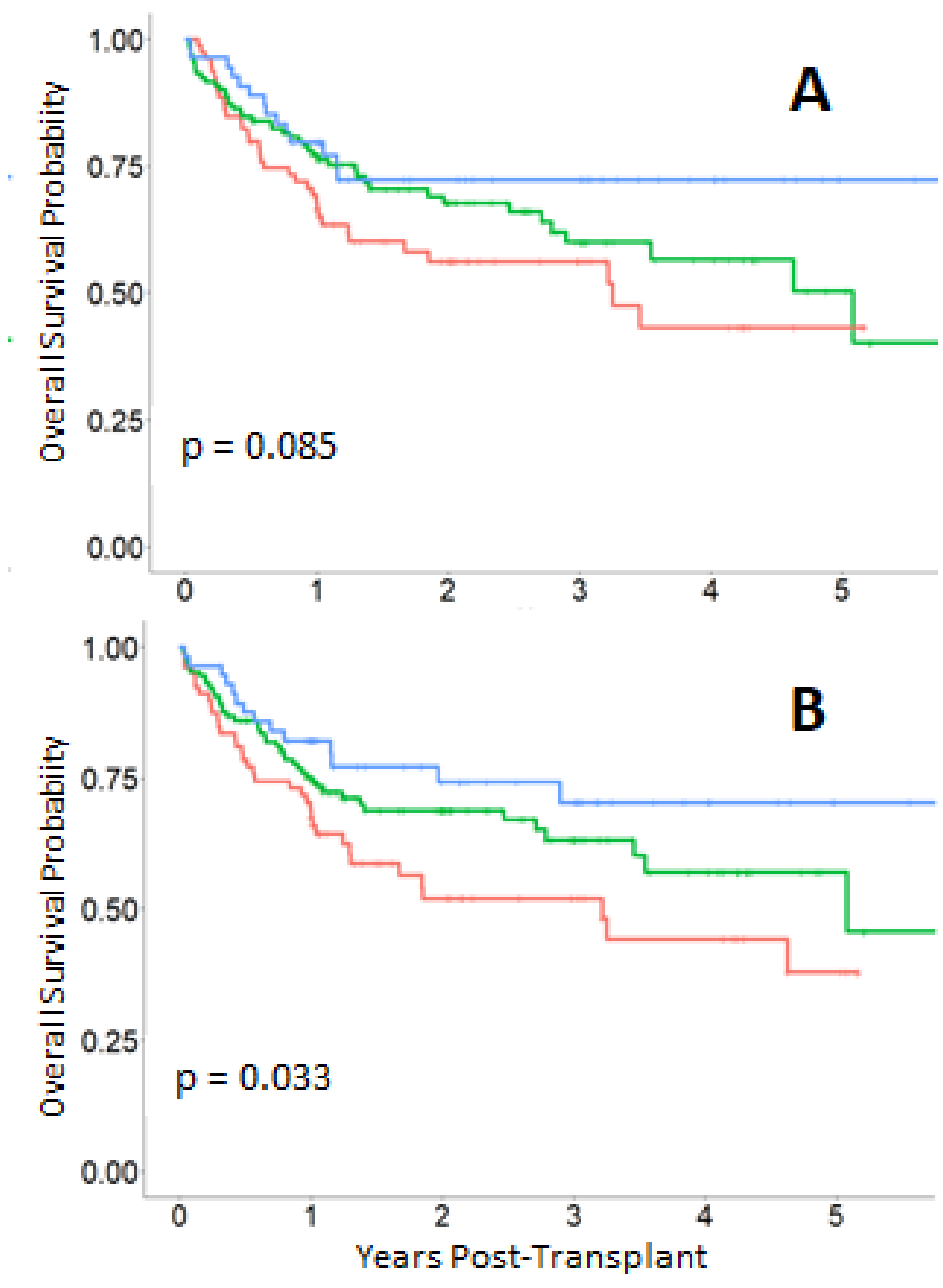

Supplemental Table 1. List of alleles not found in v2 worksheet and closest substitute allele identified

Allele count in cohort	Genotyped Allele or Ambiguity	Genotyped Allele G-group	Allele selected by worksheet	Closest allele eplets used	Antigen difference from closest allele or G-group alternative	Potential eplet difference	Comment
9	A*23:17	A*23:01	A*23:03	A*23:01	283H>P	0	1
1	A*24:02L	A*24:02	A*24:02	A*24:02	N/A	0	2
2	A*68:30	N/A	A*68:25	A*68:01	62RN>GE	0	3
4	B*15:220	B*15:03	B*15:21	B*15:03	none	0	1
1	B*40:180	N/A	B*40:18	B*40:09	114D>H, 116Y>D	1 to 4*	
1	B*40:27	N/A	B*40:26	B*40:03	116N>Y	1 to 2	
1	B*35:30	B*35:30	B*35:29	B*35:01	97R>S	1 to 2	
1	B*08:18	N/A	B*08:13	B*08:01	90A>D	1 to 2	
1	C*03:07	C*03:07	C*03:06	C*03:10	77S>N	1 to 2	
1	C*15:13	C*15:02	C*15:09	C*15:02	229E>Q	0	1
1	C*03:357	C*03:03:01	C*03:19	C*03:03	321C>Y	0	1
2	C*07:43	N/A	C*07:29	C*07:10	99S>Y	0*	1
7	C*17:03	C*17:01	C*17:02	C*17:01	none	0	3
16	C*07:18	C*07:01	C*07:13	C*07:01	324A>V	0	1
1	C*17:38	C*17:01	C*17:02	C*17:01	345A>T	0	1
2	C*15:04	C*15:04	C*15:03	C*15:09	113H>Y	0	1
3	C*04:82	C*04:01	C*04:05	C*04:01	300>InsAVL	0	1
2	C*08:06	N/A	C*08:04	C*08:03	163T>A	1 to 2	
2	C*04:06	C*04:06	C*04:05	C*04:03	156R>L	1 to 2	
2	DRB1*07:16	N/A	DRB1*07:01	DRB1*07:01	23R>G	0*	1
6	DRB1*04:92	N/A	DRB1*04:18	DRB1*04:07	207V>M	0*	1
1	DRB1*04:79	N/A	DRB1*04:18	DRB1*04:04	47Y>H	1 to 2*	
2	DRB1*11:129	DRB1*11:06	DRB1*11:11	DRB1*11:06	179S>N	0	1
1	DRB1*16:05	N/A	DRB1*16:04	DRB1*16:02	67L>I	0	3
5	DRB3*01:62	DRB3*01:01	DRB3*01:11	DRB3*01:01	N/A	0	1
2	DRB3*02:24	DRB3*02:01	DRB3*02:19	DRB3*02:01	164F>V	1 to 2	
1	DRB3*01:16	DRB3*01:01	DRB3*01:11	DRB3*01:01	170V>F	0*	1
1	DRB5*02:06	DRB5*02:02	DRB5*02:05	DRB5*02:02	98K>E	1 to 2*	
30	DQB1*03:19	DQB1*03:01	DQB1*03:13	DQB1*03:01	185T>I	1 to 2	
1	DQB1*05:08	DQB1*05:03	DQB1*05:04	DQB1*05:03	167R>H	1 to 2	
2	DQB1*03:70	N/A	DQB1*03:13	DQB1*03:03	13G>A	1 to 2*	
2	DQA1*05:08	DQA1*05:01	DQA1*05:05	DQA1*05:01	138S>I	0	1
1	DPB1*463:01	DPB1*04:02	DPB1*46:01	DPB1*04:02	96R>K, 178M>L	1 to 4	
11	DPB1*135:01	DPB1*05:01	DPB1*13:01	DPB1*05:01	205M>V	0	1

Supplemental Table 1, continued.

Allele count in cohort	Genotyped Allele or Ambiguity	Genotyped Allele G-group	Allele selected by worksheet	Closest allele eplets used	Antigen difference from closest allele or G-group alternative	Potential eplet difference	Comment
1	DPB1*131:01	DPB1*17:01	DPB1*13:01	DPB1*17:01	96R>K, 194R>Q	1 to 4	
4	DPB1*107:01	DPB1*13:01	DPB1*10:01	DPB1*13:01	N/A	0	1
6	DPB1*124:01	DPB1*03:01	DPB1*11:01	DPB1*03:01	96K>R	1 to 2	
1	DPB1*913:01	DPB1*34:01	DPB1*85:01	DPB1*34:01	96K>R, 147R>C	1 to 2*	
1	DPB1*93:01	N/A	DPB1*85:01	DPB1*20:01	57D>E	1 to 2	
2	DPB1*48:01	N/A	DPB1*47:01	DPB1*46:01	35LV>FV, 57D>E	1 to 4	
1	DPB1*125:01	N/A	DPB1*11:01	DPB1*55:01	36A>V, 69E>K, 76M>V	1 to 6*	
15	DPB1*105:01	DPB1*04:02	DPB1*10:01	DPB1*04:02	178M>L	1 to 2	
26	DPB1*104:01	DPB1*03:01	DPB1*10:01	DPB1*03:01	205M>V	0	1
3	DPA1*02:07	DPA1*02:07	DPA1*02:03	DPA1*02:02	91V>M	0	1
4	DPA1*02:12	DPA1*02:07	DPA1*02:03	DPA1*02:02	91V>M, 224R>Q	0	1
8	DPB1*05:01/ 135:01	DPB1*05:01	G-group ambiguity		205M>V	0	1
4	DPB1*04:01/ 126:01	DPB1*04:01	G-group ambiguity		178M>L	1 to 2	
1	DPB1*04:01/ 350:01	DPB1*04:01	G-group ambiguity		96K>R, 170T>I	1 to 2	
4	DPB1*04:02/ 105:01	DPB1*04:02	G-group ambiguity		178M>L	1 to 2	
2	DPB1*17:01/ 131:01	DPB1*17:01	G-group ambiguity		96K>R, 170I>T, 194Q>R	1 to 4	

* Alleles not fully sequenced for all exons could potentially harbor additional eplet differences from the assigned allele.

Comment Key: 1, Antigen difference is not part of a defined v2 worksheet eplet; 2, Expression variants are not captured by EpMM model; 3, Eplet ambiguity did not affect cohort due to eplet or allele matching between patients and donors

Supplemental Table 2: Corrected errors in v2 worksheet and rare allele eplet corrections

Class I	Error description	Correction
184H	C*17:01 listed as 184H, actually 184R	184R not a listed eplet in v2; deleted 184H from all C*17 alleles.
62GE	A*68:30 allele is not listed in v2 worksheet; closest match has 62RR.	add 62GE, delete 62RR from A*68:30 allele
17WR	not counted due to worksheet cell reference error	Edited cell reference to correct the worksheet and count 17WR.
156RA	C*04:06 allele is not listed in v2 worksheet; substituted allele has 156RA.	Corrected eplet from 156RA to 156LA for C*04:06
186R	186R incorrectly assigned to A*33:03	deleted eplet for A*33:03
177DK	Eplet not correctly listed for some alleles of B*40	delete eplet for B*40:02/03/04/05/06/08/09/11/13/18/19/20/26/27/40/50/180
211T	C*02:10 incorrectly listed has having eplet	Deleted eplet for C*02:10
76VS	C*03:07 allele is not listed in original worksheet; closest match has 76VS	Deleted eplet 76VS for C*03:07
193PV	Eplet not correctly listed for some alleles.	Deleted eplet for C*16:01/02/04
166ES	Not counted due to worksheet cell reference error	Edited cell reference to correct the worksheet and count 166ES.
**Class II**	**Error description**	**Correction**
70QT	DRB1*15:01,02,03, and some DRB3 and DRB5 not listed as 70QT	Add 70QT to DRB5*02 and DRB1*15; delete 70Q eplet.
180V	DRB1*07:01/16 not listed as 180V	Add eplet 180V to DRB1*07:01/16
16H	DRB1*07:01/16 not listed as 16H	Add 16H to DRB1*07:01/16
149H	DRB1*11:01/02/03/04/05/06 listed as 149Q; actually 149H	Changed DRB1*11:01/02/03/04/05/06 from 149Q to 149H
57A	DRB1*14:54 listed as 57D; actually 57A	Changed DRB1*14:54 from 57D to 57A
178L	DPB1*105:01 was assigned closest allele; actually has 178L, not 178M	Changed 178M to 178L for DPB1*105:01
96R	DPB1*463:01 was assigned closest allele; actually has 96K, not 96R	Changed 96K to 96R for DPB1*463:01
35FV	DPB1*48:01 was assigned closest allele, actually has 35LV, not 35FV.	Changed DPB1*48:01 from 35FV to 35LV (use column U for 35LV).
56EE	DPB1*48:01 was assigned closest allele, actually has 57D, not 56EE.	Changed DPB1*48:01 from 57D to 56EE
185T	DQB1*03:19 was assigned closest allele; has 185I, not 185T.	Changed DQB1*03:19 from 185T to 185I
130Q	DQB1*06:11 listed as 130q; actualy 130R	Changed DQB1*06:11 from 130q to 130R
96H	Inconsistent use of 96HK, 96H, and 98K. 96HK is both 96H and 98K.	Delete 96HK for DRB1*07:01/16, 09:01/02, DRB3; added 96H and 98K to all 96HK alleles in DRB1*3/8/11/12/13/14
96EV	DRB5*02:06 was assigned closest allele; actually does not have 96EV	Deleted 96EV for DRB5*02:06; also replaced 98K with 98E.
70D	v2 worksheet improperly excluded from antibody verified section without correcting in "other" section (similar to 166ES in class I). Has since been verified. Add to worksheet	Edited cell reference to properly count 70D.
65L	DPB1*93:01 assigned 65L; change to 65I.	DBB1*93:01 changed from 65L to 65I
69E	DPB1*93:01 listed as 69K; actually 69E. DPB1*125:01 listed as 69E; actually 69K	Made appropriate edits to DPB1*93:01 and DPB1*125:01
96K	DPB1*131:01 listed as 96R, actually 96K. 124:01 listed as 96K, actually 96R.	Made appropriate edits to DPB1*124:01 and 131:01
26L/Y/F	Due to an error in the worksheet logic, 26L, 26Y, and 26F are not properly counted.	Edited cell reference to correct the worksheet and count 26L/Y/F.

Supplemental Table 3: Univariate association of patient, donor, and transplant demographics with outcomes.

Outcome	Overall Survival	Relapse - Free Survival	Relapse	Non-relapse mortality	Acute GvHD II-IV	Acute GvHD III-IV	Chronic GvHD	Chronic GvHD - extensive	Neutrophil Engraftment	Platelet Engraftment
Numerical variable	p-value of wald test									
patient age	**< 0.0001**	**0.0003**	0.9	**< 0.0001**	0.11	0.83	0.81	0.89	**0.0052**	**< 0.0001**
performance value	**0.0003**	**0.0001**	**0.03**	**0.013**	0.84	0.89	0.34	0.36	**0.035**	**< 0.0001**
Donor age	0.69	0.79	0.97	0.7	0.13	0.58	0.054	**0.02**	0.59	0.78
CD34+ dose	0.96	0.83	0.47	0.64	0.69	0.21	**0.003**	**0.008**	0.2	0.46
Categorical variable	p-value of log-likelihood test									
patient sex	0.51	0.99	0.94	0.86	0.99	0.87	0.56	0.1	0.93	0.25
patient race group (5 groups)	0.22	0.37	**0.033**	**0.046**	0.34	0.72	0.33	0.29	**0.01**	0.14
primary malignancy category	0.87	0.79	0.13	0.69	0.98	0.31	0.24	0.27	0.62	0.14
DRIscore (Low-Mod v. High/v.high)	**0.0002**	**< 0.0001**	**0.0002**	**0.026**	0.17	0.68	0.8	0.35	0.058	**0.0073**
HCT-CI > 0	**0.0077**	**0.0027**	0.19	**0.018**	0.11	0.29	**0.041**	**0.011**	0.92	0.79
Bone marrow vs PBSC	0.48	0.85	**0.029**	**0.0061**	0.88	0.062	**0.027**	0.16	0.56	0.73
CD3+ dose > 1.77 x 10^8/kg	0.8	0.72	0.31	0.64	0.39	**0.0057**	0.06	**0.0091**	0.8	0.27
Donor Sex	0.85	0.9	0.35	0.45	**0.019**	0.96	0.15	0.51	0.64	0.89
Gender mismatch	0.89	0.66	0.74	0.38	**0.0072**	0.64	0.21	0.74	0.66	0.75
ABO type mismatch	0.45	0.51	0.12	0.62	0.82	0.47	0.35	0.3	0.97	0.88
CMV serostatus	0.42	0.15	0.25	0.74	0.63	0.38	0.81	0.43	0.63	0.13
conditioning intensity: RIC/NMA vs MA	**0.002**	**0.013**	0.93	**0.0046**	0.073	0.44	0.086	**0.022**	**0.0013**	**0.0003**
GvHD prophylaxis (Tacrolimus vs Sirolimus)	0.74	0.93	0.9	0.91	0.39	0.14	0.99	0.9	0.86	0.59
Transplant year (2011-2016 vs 2017-2020)	0.9	0.39	0.1	0.79	0.79	0.18	**0.03**	0.07	0.44	0.21
CHIME (0 vs. 1 vs. 2)	**< 0.0001**	**0.0003**	0.33	**0.0011**	0.063	**0.049**	0.67	0.94	0.37	0.26

Supplemental Table 4: Median EpMM by Ethnicity, HLA Class, and Mismatch Vector

	Black	Hispanic	Asian	Native Hawaiian/ Pacific Islander	White (non-Hispanic)	Kruskal-Wallis rank sum p
HvG Class I	10	10	9	11.5	11	0.71
GvH Class I	9	11	9	5.5	12	0.023
HvG Class II	21.5	24	24	6	22.5	0.24
GvH Class II	26.5	25.5	24	52	24.5	0.46

Supplemental Table 5: Incidence of Mortality and Relapse for Combinations of CHIME and CD3 dose.

Specific Mortality and Relapse Outcomes	CHIME 0 or CHIME 1 with low CD3	CHIME 2 or CHIME 1 with high CD3	OR (95% CI)	p
Relapse - free survival	85	67	0.50 (0.29 - 0.85)	0.008
Survival with clinical relapse	9	6	0.62 (0.18 - 2.02)	0.43
Death from Clinical Relapse	20	21	1.00 (0.49 - 2.07)	1
Death from GvHD	2	12	6.16 (1.33 - 57.8)	0.011
Death from Infection	8	16	2.03 (0.78 - 5.70)	0.14
Death from Organ Dysfunction	2	10	5.05 (1.05 - 48.4)	0.035
Death from unknown cause	1	1	n.s	

n = 260; Graft CD3 data for 5 patients in CHIME 1 category were missing.
